# Identification of Quantitative Trait Loci for Altitude Adaptation of Tree Leaf Shape With *Populus szechuanica* in the Qinghai-Tibetan Plateau

**DOI:** 10.3389/fpls.2020.00632

**Published:** 2020-05-27

**Authors:** Meixia Ye, Xuli Zhu, Pan Gao, Libo Jiang, Rongling Wu

**Affiliations:** ^1^Beijing Advanced Innovation Center for Tree Breeding by Molecular Design, Beijing Forestry University, Beijing, China; ^2^Center for Computational Biology, College of Biological Sciences and Technology, Beijing Forestry University, Beijing, China; ^3^Center for Statistical Genetics, The Pennsylvania State University, Hershey, PA, United States

**Keywords:** leaf shape, altitude, QTL, *Populus szechuanica*, Qinghai-Tibetan Plateau

## Abstract

As an important functional organ of plants, leaves alter their shapes in response to a changing environment. The variation of leaf shape has long been an important evolutionary and developmental force in plants. Despite an increasing amount of investigations into the genetic controls of leaf morphology, few have systematically studied the genetic architecture controlling shape differences among distinct altitudes. Altitude denotes a comprehensive complex of environmental factors affecting plant growth in many aspects, *e.g.*, UV-light radiation, temperature, and humidity. To reveal how plants alter ecological adaptation to altitude through genes, we used *Populus szechuanica* var. *tibetica* growing on the Qinghai-Tibetan plateau. *F*_ST_ between the low- and high- altitude population was 0.00748, *Q*_ST_ for leaf width, length and area were 0.00924, 0.1108, 0.00964 respectively. With the Elliptic Fourier-based morphometric model, association study of leaf shape was allowed, the dissection of the pleiotropic expression of genes mediating altitude-derived leaf shape variation was performed. For high and low altitudes, 130 and 131 significant single-nucleotide polymorphisms (SNPs) were identified. QTLs that affected leaf axis length and leaf width were expressed in both-altitude population, while QTLs regulating “leaf tip” and “leaf base” were expressed in low-altitude population. *Pkinase and PRR2* were common significant genes in both types of populations. Auxin-related and differentiation-related genes included *PIN1, CDK-like*, and *CAK1AT* at high altitude, whereas they included *NAP5, PIN-LIKES*, and *SCL1* at low altitude. The presence of S*tress-antifung* gene, *CIPK3* and *CRPK1* in high-altitude population suggested an interaction between genes and harsh environment in mediating leaf shape, while the senescence repression-related genes (*EIN2* and *JMJ18*) and *JMT* in jasmonic acid pathway in low-altitude population suggested their crucial roles in ecological adaptability. These data provide new information that strengthens the understanding of genetic control with respect to leaf shape and constitute an entirely novel perspective regarding leaf adaptation and development in plants.

## Introduction

Leaves are the site of plant photosynthesis and are important for both water balance and temperature adjustment. Considering these essential functions, leaf shape is an important factor by which plants maximize their survival (Nicotra et al., 2008; Moon and Hake, 2011). In response to varying geography and climate, plant genomes must undergo modification to develop favorable leaf shapes (Nicotra et al., 2011). As an influential environmental factor, altitude can significantly alter variation of plants’ ecological adaptability and result in the change in leaf morphology (Milla and Reich, 2011), because it reflects a complex combination of factors (e.g., temperature, water, sunlight, and soil fertility). It has been widely accepted that altitude-determined morphogenetics are of great ecological and evolutionary importance for studying mechanisms of shape variation and biological adaptability (Klingenberg, 2010).

To compare the effects of altitude, using the oak metapopulation, Firmat et al. (2017) recently investigated the evolutionary, genetic, and environmental components of the timing of leaf unfolding and senescence along an elevation gradient. In seven successive elevation gradients on the Qinghai-Tibetan plateau, Lu (2017) revealed relationships among leaf characteristics in *Halenia elliptica*. The study revealed gradual alteration of leaf shape, which was accompanied by changes in photosynthetic pigment content, osmotic adjustment, and the anti-oxidation system, suggesting that an active stress response caused leaf shape alteration. Along global elevational gradients, Midolo et al. (2019) quantified changes in seven morpho-ecophysiological leaf traits in 109 plant species on four continents. Although altitude is presumed to influence leaf shape variation, the genetic mechanism mediating the effect of altitude on leaf shape has been elusive. Using molecular approaches, the respective leaf shape genes *ANGUSTIFOLIA* (*AN*), *ROTUNDIFOLIA3* (*ROT3*), and *REDUCED COMPLEXITY* (*RCO*) were revealed to regulate leaf width, length, and affect developing leaflets by repressing growth at their flanks respectively (Tsukaya, 2006; Bai et al., 2013; Vlad et al., 2014; Koyama et al., 2017). A module of auxin-*PIN1*-*CUC2* was discovered to affect function in patterning leaf margin (Bilsborough et al., 2011). However, the studies characterizing genes responsible for shape morphology have identified their roles in the context of development; thus, relationships between shape genes and elevation-caused ecological changes have not yet been established.

As one of the largest plateaus worldwide, the Qinghai-Tibetan plateau is a cradle of the planet’s natural wealth on the earth. The high altitude of the Qinghai-Tibetan plateau has low temperatures, high UV radiation, and poor-quality soils, which combine to provide a unique natural laboratory for studying adaptability and evolution (Li and Fang, 1999). Trees growing on the Qinghai-Tibetan plateau face a variety of abiotic stresses; therefore, it is likely that they have been subjected to a series of adaptive or evolutionary changes. Genotypes are presumed to differ with altitude, including differences in leaf morphology. When performing genetic analyses, leaf shape is regarded as a quantitative phenotypic trait (Klingenberg, 2010). For quantitative genetic purposes, the characterization of leaf morphological variations at different altitudes is important for understanding a plant’s long-term strategies for adaptation to environmental pressure (Miles and Ricklefs, 1994). The complex combination of climatic factors, as well as the extensive geographical diversity at different elevations, could have significant potential for the development of inheritable leaf morphological variation. Therefore, the association study was performed using natural populations from different altitudes in this study; this approach offers a compelling opportunity to dissect the genetic control of altitudinal expression on leaf morphology. This work can aid in elucidating connections among shape gene knowledge, ecological adaptation, and evolution.

For mapping leaf shape QTLs, leaf width, length, and angle were added to the conventional statistical model (Tian et al., 2011). To capture precise leaf shape information, leaf outlines were used based on the geometric morphometrics technique (Bookstein, 1991; Slice, 2007). The Procrustes alignment method is an accurate and lossless shape information method that filters out positional, sized, and rotational effects on leaf shape; it can fully encode all information that cumulatively influences leaf shape contouring. The radius-centroid-contour method uses the length curve of the radius from leaf centroid to all outline landmarks to obtain the minute contours of shape (Fu et al., 2013). A method uses the Elliptic Fourier (EF) series to enable accurate measurement of an object’s outline had been developed recently, which has the inherent advantage of using sparse parameters to describe shape (Neto et al., 2006; Bo et al., 2014). The combination of genetic mapping and EF-based geometric morphometrics supported the development of “shape mapping,” which led to the construction of *hp*QTL, a computational platform for mapping heterophylly QTL (Sun et al., 2018). The development of “shape mapping” has enabled genetic analysis of the effect of altitude on leaf shape.

To study the altitude-specific variation pattern of leaf morphology, this study used leaves from *Populus szechuanica var. tibetica*, a common indigenous tree species naturally distributed in the Qinghai-Tibetan plateau of China. The large elevation distribution on the Qinghai-Tibetan Plateau (ranging from 1100 to 4500 m, Shen et al., 2014), coupled with the distribution of *P*. *szechuanica* throughout, makes this species a useful model for studying ecological adaptability and leaf shape evolution. The objectives of this study were to (1) use the EF-based shape model to identify QTLs controlling the overall shape differences between low and high altitudes; (2) determine how altitude influences the genetic factors underlying leaf shape; and (3) establish a link between altitude and the expression of leaf shape QTLs. By analyzing leaf shape QTLs of *P*. *szechuanica* at different elevations, our work identified key QTLs that relate to leaf shape variation and ecological evolution; the results offer new opportunities and novel instrumentation for dissecting complex traits from the perspective of ecological evolutionary developmental biology.

## Materials and Methods

### Mapping Population

*Populus szechuanica*, one of the *Cathay poplar* group of species in the genus *Populus*, is primarily distributed throughout the Qinghai-Tibetan Plateau, as well as the Sichuan and Yunnan Provinces in China. Leaves from trees representing different elevational populations of *P*. *szechuanica* were sampled in the Sejila mountain range, a provenance site in the Nyingchi prefecture of the Tibet Autonomous Region. A total of 119 individual trees from high altitude (2600–3090 m above sea level) and 141 individual trees from low altitude (2000–2300 m above sea level), as shown in Supplementary Figure S1, were sampled to construct the mapping population. In July 2012, 5–10 branches with vigorous growth and thick wood were cut from each tree and were stored in sand. After 2 years of extensive propagation in a common nursery (Dayi county, Sichuan Province), new cuttings of uniform length and diameter were planted in a common garden experiment in 2015. A mean of ≥10 clonal replicates were made for each tree from each population. This created a clonal line for each of the trees.

### Phenotyping and Genotyping

For each clone from each original tree, one or two from each of the upper, middle, and lower sections of the crown of each clonal tree were sampled to measure leaf shapes. In total, 5,819 leaves from lines of the high-altitude group and 10,628 leaves from lines of the low-altitude group were collected and photographed to analyze their shapes. Digital images were taken of each leaf using a pose-fixed camera. The resulting RGB pictures of the leaves were converted to binary shape images, with white color representing the leaf area and black color denoting the image. Using a shape alignment method described by Fu et al. (2013), differences in the location and rotation of leaf position in the images, as well as differences in the effect scale due to leaf size, were removed using an orthogonal Procrustes method (Gower and Dijksterhuis, 2004). Differences in the shapes of leaves from clones in a given line could occur due to variations in the micro-environment (i.e., differences within nursery blocks) or due to heterophylly, which could cause leaves from different sections of the tree to have different shapes (Winn, 1999). To adjust for the potential influences of these factors, leaves from all sections of the clones in each line were used to derive an average shape by calculating the arithmetic average of each coordinate, along with several contour landmarks. The appropriate number of landmarks was determined using the Akaike Information Criterion for all 260 average shapes from plants of both altitude groups. The use of 70 landmarks had the lowest Akaike Information Criterion value. Therefore, coordinates of 70 landmarks along the boundary of each average leaf shape for each line, which had equal radii, were used as the phenotype for further mapping of leaf shape.

For all 260 lines, a genome-wide panel of single-nucleotide polymorphisms (SNPs) was sequenced by re-sequencing technology using an Illumina Hiseq^TM^2000 platform. Each sample was deep sequenced to approximately 20-fold to generate a total of 13.71 billion raw paired-end reads. After removal of low-quality reads by filtering, the remaining clean reads were aligned against a reference genome of *Populus trichocarpa*,^1^ deriving a total of approximately 12.76 million high-quality SNPs. After sequencing and the removal of low-quality sequence, workflow consisting of 3 major steps was employed: mapping, improvement and variant calling. For mapping, BWA-0.7.5^2^ was firstly used to prepare the Burrows Wheeler Transform index for the reference using the index command, the MEM mode was applied to align reads to genome, with *-R* paramter designating the reference. SAMTOOLS-1.9 version^3^ used command of fixmate and sort to order the mapped sequence into coordinate order within BAM file. For improvement step, all BAM files from each sample were merged and indexed respectively using the merge and index function. For the variant calling step, bcftools mpileup command was used to convert BAM into BCF file, which contained all genomic positions, using *-Ob -f -vmO z* parameter. Variant filtration was performed using bcftools filter to derive the final VCF file to recode the genotype at genome-wide level, parameter of *-s LOWQUAL -i ‘%QUAL>20’* were set to seperate true SNPs from error SNPs. Using Vcftools (Danecek et al., 2011), SNPs with a sequencing depth greater than 12, *p*-value of Hardy Weinberg equilibrium test greater than 0.05, minor allele frequency greater than 5%, and a missing rate and heterozygosity less than 10% were screened for further association studies. These criteria were satisfied by a set of 81,839 SNPs. For natural population, some SNPs segregated into 2 genotypes, still there were SNPs segregated into 3 genotypes, but their segregation ratio do not necessarily follow 1:1 or 1:2:1. Nonetheless, we use “testcross” and “intercross” to refer to these segregation types, but without considering the segregation ratio. SNPs of intercross type had 2 homozygous genotypes and 1 heterozygous genotype in natural population, while the testcross SNPs had 1 homozygous and 1 heterozygous genotype. All SNPs included 53,094 intercross markers and 28,745 testcross type of markers. The genotyping data in a format of VCF file were submitted to the European Variation Archive,^4^ with a project ID of PRJEB36028.

### Population Structure and Calculation of *F*_ST_ and *Q*_ST_

FastStructure (Raj et al., 2014) was used to infer population structure from the large SNP dataset. Combining individual genotypes from both high- and low- altitude, this software identified *K = 2* as the optimal group number for the whole population, as likelihood peak can be seen for *K* = 2 from Supplementary Figure S2A. All 260 genotypes can be divided into 2 subgroups. The high-altitude contained 97 and 22 genotypes, forming sub-population 1 and 2, the low-altitude population contailed 9 and 132 genotypes for sub-population 1 and 2 (Supplementary Figure S2B). Therefore, a total of 106 and 154 genotypes constituted the “real high altitude population” and “real low altitude population,” with the high- and low-altitude provenance occupying the ratio of 97/106 and 132/154. Further association study of shape was based on the two adjusted sub-populations.

With the normalized morphological shapes, we derived the detailed shape trait of leaf length, leaf width and leaf area using the home-made R script. Analysis of variance (ANOVA) were performed to calculate the within-deme variance ($\sigma_{G\,W}^{2}$) and between-deme variance ($\sigma_{G\,B}^{2}$) for each trait. Equation of $Q_{S\,T}=\sigma_{G\,B}^{2}/\left(\sigma_{G\,B}^{2}+2\,\sigma_{G\,B}^{2}\right)$ was employed to *Q*_ST_ values with the 3 traits (Raeymaekers et al., 2007). From all 81,839 SNPs, we selected 68867 neutral SNPs using their genomic position information, comprising 53063 SNPs locating at intergenic DNA sequence, 15575 SNPs residing at intron region, and 229 synonymous SNPs at exon region. Based on neutral SNPs, *F*_ST_ were calculated using the diveRsity package with R language (Keen et al., 2013).

### ANOVA Analysis of Shape Traits

To test the significance effect of altitude, ANOVA analysis was performed using descriptor of shape, e.g., leaf length, leaf width and leaf area. For each of the trait, the phenotypic value for leaf *l* of genotype *i* from the altitude *j* from tree position *k* under block *m* is expressed as *y*_*l*|*ijkm*_ = μ + α_*i*_ + *g*_*j*|*i*_ + *p*_*k*_ + β_*m*_ + *GPI*_*jk*|*i*_ + *API*_*ik*_ + ε_*ijklm*⁣⋅_⋅α_*i*_ is the *i*th altitude effect, *g*_*j—i*_ is the genetic effect due to the *j*th progeny nested in altitude *i*, *p*_*k*_ is the position effect due to the *k*th position on the tree from upper to lower, β_*m*_ is the block effect due to the *m*th block within the filed of common garden. *GPI*_*jk—i*_ is the interaction effect between the *j*th genotype and *k*th position given the *i*th altitude. *API*_*ik*_ is the interaction effect between the *i*th altitude and *k*th position. ε_*ijklm⋅*_is the residual. The ANOVA analysis was performed using *aov* function in R language.

### Framework of the Shape Association

The overall population was presumed to contain *n*_*H*_ individuals from high altitude and *n*_*L*_ individuals from low altitude, all of which were genotyped for a panel of genome-wide SNPs. Based on geometric morphometrics, the *x*- and *y*-coordinate sequences of shape were used to represent the phenotypic data. The average shape for each line was calculated. For each average leaf, a total set of *K* landmarks were sampled clockwise at equal angle intervals along the margin. Let (*x*_*Hi*_,*y*_*Hi*_) = (*x*_*Hi*_(1),*y*_*Hi*_(1);*x*_*Hi*_(2),*y*_*Hi*_(2);…;*x*_*Hi*_(*K*),*y*_*Hi*_(*K*)) denote the sequence of *x*- and *y*-coordinates of *K* landmarks for individual *i* in the high-altitude population. Let (*x*_*Li*_,*y*_*Li*_) = (*x*_*Li*_(1),*y*_*Li*_(1);*x*_*Li*_(2),*y*_*Li*_(2);…;*x*_*Li*_(*K*),*y*_*Li*_(*K*)) denote *x*- and *y*-coordinates of *K* landmarks for individual *i* from the low-altitude population. For a given QTL carrying *J* genotypes, *e.g., QQ*, *qq*, and *Qq*, the set of landmarks enabled the construction of the likelihood using multivariate normal distribution. By referring to geometric methods (Bo et al., 2014), mean vector within density probability of multivariate normal distribution, equivalent to the shape descriptor, was modeled by Elliptic Fourier function, the order with which can be determined by an AIC criterion. The covariance matrix within the multivariate normal distribution was modeled by using an autoregressive model to structure the spatial (co)variance among all *K* outline landmarks (Zhao et al., 2005). In such framework, *H*_0_ was proposed as no shape difference existed between genotypes, *H*_1_ was proposed as the opposite. The test statistic for this hypothesis was calculated as the log-likelihood ratio (LR) of the null against the alternative model. The LR threshold was determined by an empirical approach based on 1,000 replications of permutation tests to derive significant SNPs (Churchill and Doerge, 1994). The model was implemented using R language, the code has been attached as an Supplementary File S1 of Presentation 1.ZIP. The association of leaf area was performed using TASSEL software (Zhang et al., 2010).

## Results

### Shape Phenotype

**FIGURE 1:**
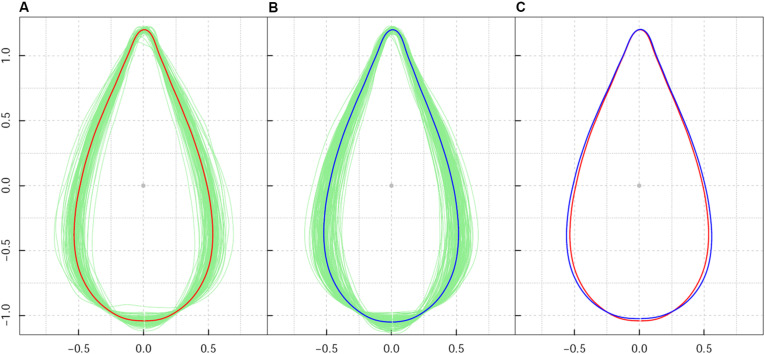
Shape pattern of *Populus szechuanica* var. *tibetica* leaves sampled from high and low altitudes in the Qinghai-Tibetan plateau. **(A)** Denotes the mean shape of all 119 leaves from high-altitude populations; **(B)** denotes the mean shape of all 141 leaves from low-altitude populations; **(C)** shows the comparison of the mean shape of leaves at the population level. Both shapes from high- and low-altitude populations were corrected by Procrustes alignment.

While it differed from the direct collection of leaves from trees in their natural growing environments, the design of a common garden plantation ensured a uniform environment and consistent management of plant growth. As a result of the homogeneous growth conditions in the plantation nursery, leaf shape diversity within the sample population reflected the intrinsic effect of genetic background on leaf shape. After calculation of average leaf shape, all Procrustes-aligned shapes (Figures 1A,B) had consistent orientations in both high-altitude and low-altitude populations. Leaf shape centroids with all 260 lines overlapped to the origin of the coordinate system. The absence of extraordinarily sized leaf shapes indicated a clear effect of Procrustes alignment in leaf shape association.

Among all samples, the average leaf shape varied from ovate-orbicular to ovate-lanceolate. As shown in Figures 1A,B, leaf shape variation for *P*. *szechuanica* was precisely confirmed based on leaf axis length, leaf width, length to width ratio, and the region where the broadest region of the leaf was located. For leaf axis length specifically, longer leaf shapes typically possessed a less-broad blade base. The broadest region of most leaves was approximately 1/4–1/3 of the length from the blade base. Relatively speaking, high-altitude plants yielded a slender and slightly narrowed blade base, whereas shorter leaves within the low-altitude population had greater width in the middle-lower section of the leaf shape (Figure 1C). Statistical analysis revealed that high-altitude leaves showed a leaf width variance of 0.00906 and leaf axis length variance of 0.000739. Low-altitude leaves showed a 1.386-fold change in width variance, compared with that from high-altitude leaves; the on-axis length variance was similar (0.000833).

### ANOVA of Variation on Quantitative Morphology of Leaf

Outline points along leaf boundary for describing leaf morphology involve complex statistical issue in dissecting its variance source, therefore, we switch to use other descriptor of shape, e.g., leaf length, leaf width, leaf area. To test the significance effect of altitude, *F-value* (Table 1) for altitude effect was 105.834, 107.570, and 117.366 respectively for trait of leaf width, leaf length and leaf area, all of which had reached to the extremely significant level. However, the statistics of *F-value* for altitude were smaller than that of leaf position of growth on the tree, suggesting the physiologically determined heterophylly of plant had larger effect than that caused by altitude.

**TABLE 1:** Analysis of variance for altitude and genotype effects on leaf shape.

Trait	Source	*F*	*p-value*
Leaf width	Altitude	105.834	<2e-16
	Genotype	52.807	<2e-16
	Block	0.815	0.367
	Leaf position	150.442	<2e-16
	Genotype × Position	0.987	0.617
	Altitude × Position	16.300	4.51e-16
Leaf length	Altitude	107.570	<2e-16
	Genotype	33.042	<2e-16
	Block	0.194	0.6596
	Leaf position	159.966	<2e-16
	Genotype × Position	1.060	0.0838
	Altitude × Position	14.514	3.24e-14
Leaf area	Altitude	117.366	<2e-16
	Genotype	46.323	<2e-16
	Block	0.217	0.6412
	Leaf position	141.837	<2e-16
	Genotype × Position	1.078	0.0373
	Altitude × Position	12.523	3.71e-12

Also, to test the overall genotype effect on leaf shape respectively nested in different altitude, the integrated model for dissecting the variance component resulted in an extremely significance of genotypic effect. Besides the similar result of significance with heterophylly effect, we observed that block had non-significant effect on shape, suggesting the clones for distinct genotype in the population didn’t show a significant difference on shape development. Importantly, the 3 leaf traits had enlarged *F-value* of interaction effect between altitude and leaf position, than that of genotype-by-position, indicating the potential necessity to explore the force of high-and low- altitude in evolving ecological adaptation.

### *F*_ST_ Value, *Q*_ST_ Value and Their Comparison

Using the neutral SNPs from the genome-wide SNP panel, *F*_ST_ value for genetic divergence was calculated. *F*_ST_ result between the high- and low- altitude population was 0.007484551, showing a very low degree of genetic divergence due to the altitude (Pujol et al., 2008). Using *F*_ST_ value, we further derived a value of 33.152 with *Nm*, using the equation provided by Wright (1951) that *Nm = (1 - Fst)/(4Fst).* This high measure had accounted for the important role of gene flow in replacing genetic drift for preventing population divergence. The combination of high gene flow and the low value of *F*_ST_ suggested that, altitude had not granted a geographical separation for population divergence along the mountain. *Q*_ST_ value for leaf width, leaf length and leaf area were calculated as 0.009243, 0.0110829, 0.009649 respectively. All *Q*_ST_ with the three traits were larger than the *F*_ST_ value, suggesting there might exist the local adaptation between the two sub-populations to cause the shape difference of leaf.

### Mapping Shape QTLs in High- and Low-Altitude Populations

To determine the optimal number of EF harmonics, the goodness-of-fit of EF was analyzed for each harmonic order, ranging from 0 to 6. As shown in Supplementary Figure S3, with increased harmonic order, the superimposed ellipses improved the goodness-of-fit with leaf shape. Harmonics 1–6 explained 53.7, 91.3, 94.9, 97.2, 98.4, and 98.8% of leaf shape information, respectively. Specifically, the 97.2% of total leaf shape variation explained by harmonic 4 exceeded 95%. Therefore, harmonic 4 was the optimal order to approach leaf shape vectors, as it was capable of providing sparse parameters for QTL mapping, laying a solid foundation for EF-based shape mapping.

**FIGURE 2:**
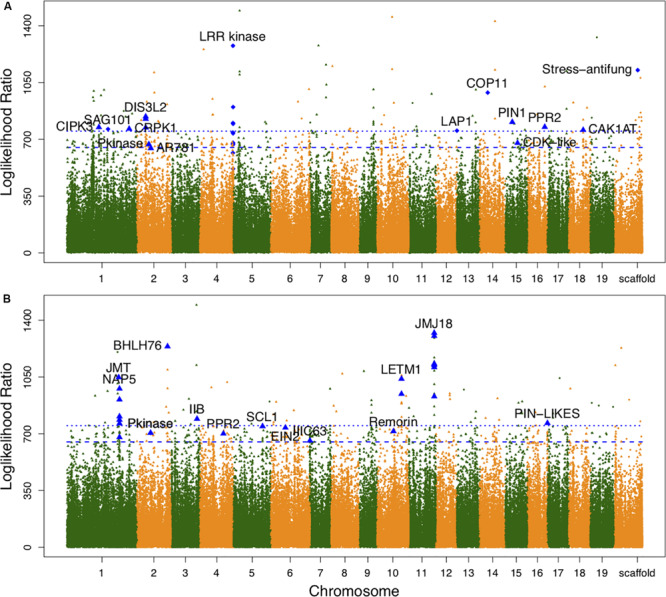
Manhattan plots of leaf shape associations for high- and low-altitude populations. **(A,B)** Are results for high- and low-altitude populations, respectively. Diamond-shaped dots denote the testcross SNPs and triangle-shaped dots represent intercross SNPs. The broken line and dotted line indicate threshold values for testcross and intercross SNPs, respectively, which were determined by 1000 replicates of permutation tests.

Associations between each SNP and all average shapes provided a plot of log-likelihood ratios, reflecting the significance of association degree (Figure 2A). As a result of the segregation of different types with SNPs, we determined the threshold values for testcross and intercross SNPs by using a permutation test (Churchill and Doerge, 1994). One hundred and thirty SNPs were determined to significantly regulate leaf shape variation in the high-altitude population; these were sporadically distributed throughout the genome. Nine of 54 testcross SNPs and 28 of 76 intercross SNPs were annotated to functional genes. Supplementary Table S1 lists detailed information regarding these SNPs, including their chromosomal position, segregation type, and functional annotation. Of the annotated SNPs, 6 highly linked SNPs that were collectively located at *LEUCINE RICH REPEAT* (*LRR*) *KINASE* genes on chromosome 4 were found to show association peaks. From chromosome 15, the SNPs within the *PIN-FORMED* (*PIN1*) gene and *CYCLIN-DEPENDENT KINASE* (*CDK*)-*LIKE* gene were found to be significant. Similar to the identification of the *CDK-LIKE* gene, one SNP within the *CDK*-*ACTIVATING KINASE* gene (*CAK1AT*) was mapped from chromosome 18. From chromosome 1, *CBL-INTERACTING PROTEIN KINASE 3* (CIPK3) and *COLD RESPONSIVE PROTEIN KINASE 1* (CRPK1) were identified. One significant SNP on chromosome 14 and on the scaffold were annotated as *COP11* and “Stress-antifung.”

For the low-altitude population, the log-likelihood ratio plot showed 131 significant SNPs (Figure 2B). Based on their functional annotations (Supplementary Table S2), we determined common annotations of *Pkinase* and *PPR2*. Although the genomic positions of *PPR2* and *LETM1* differed between the high- and low-altitude populations, the co-occurrence of these genes in both populations suggests that they have similar roles in regulating leaf shape. For clustered distribution of significant SNPs at chromosomes 1 and 11, we identified *NON-INTRINSIC ABC PROTEIN 5 (NAP5)*, *JUMOJI DOMAIN-CONTAINING PROTEIN 18* (*JMJ18)*, and *JASMONIC ACID CARBOXYL METHYLTRANSFERASE* (*JMT)*. *BHLH76*, *IIB*, and *IIIC63* belonging to transcription factor genes also regulated leaf morphology at low altitude. The organ differentiation-related gene *SCARECROW-LIKE1* (*SCL1)*, auxin transport gene *PIN-LIKES*, and *ETHYLENE-INSENSITIVE PROTEIN* (*EIN2)* were also found to include significant SNPs. The large difference in annotation results revealed different genetic architecture controlling leaf morphology between the two altitudes.

### Association of Leaf Area and Heritability

**FIGURE 3:**
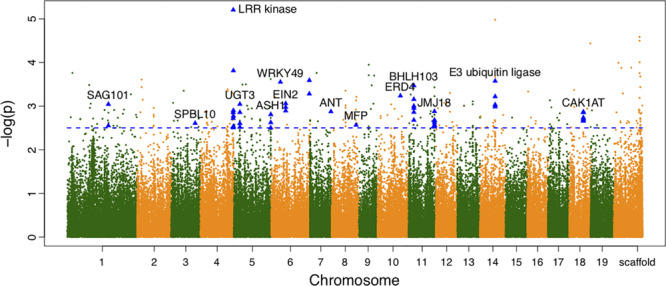
Manhattan plots of leaf area associations for the combined population. Triangle-shaped dots represent significant SNPs within the functional important genes.

We also calculated the shape area by using the normalized leaf shapes among all 260 lines. The *Q+K* model for association analysis of leaf area for the combined population resulted in 279 significant SNPs. As shown in Figure 3, 130 out of them were annotated to 78 different genes. As compared to genes discovered in result of shape association, 5 genes overlaped with the two sets, e.g., the *SAG101*, *LRR kinase, EIN2*, *JMJ18*, *CAK1AT*, suggesting the overlapping role of shape in affecting area. Nevertheless, the rest genes labeled in the manhattan plot of area trait can further emphasize the other physiological regulation in determining leaf area.

From the variance component dissection with the association model, for significant SNPs, the cumulative heritability for leaf area was 6.916%. The maximum heritability value of single SNP was observed on 18786027 *bp* of chromosome 4, explaining 6.652% variance of the area variation. Similarly, the broad-sense heritability for leaf width and leaf length was 0.263 and 18.38%.

### Types of QTL Expression

**FIGURE 4:**
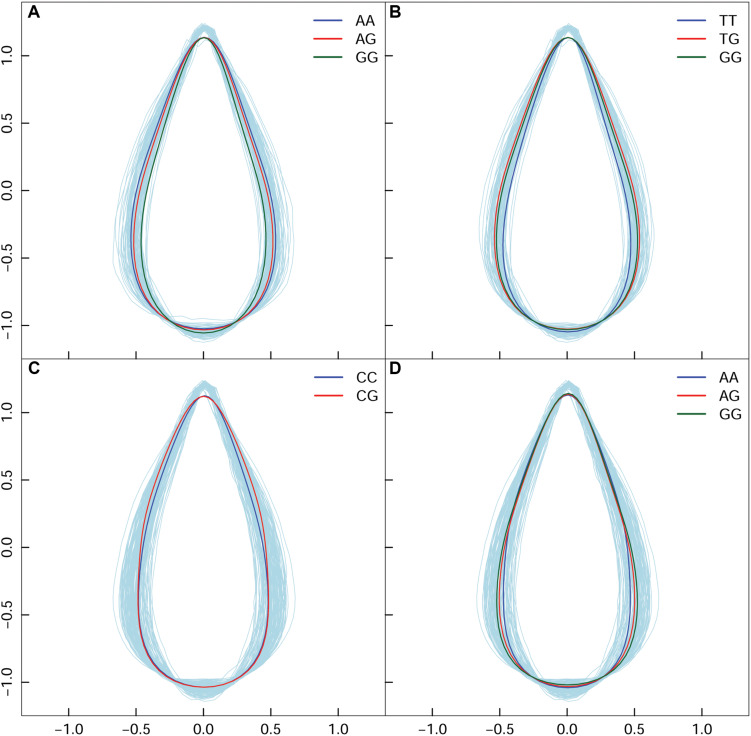
Four QTL shape patterns in both high- and low-altitude populations. **(A,B)** Represent the high-altitude-specific “leaf length” and “leaf width” QTL. **(C,D)** Represent the low-altitude-specific “blade tip” and “blade base” QTL.

In high altitude population, by calculating leaf width, length, and area, we found 130 SNPs in the high-altitude population that showed an average change of 9.18% based on width between allelic genotypes; however, an allelic average change of 0.77% was revealed based on the axis of leaf length. A correlation of 0.9496 was identified between leaf area and leaf width—this indicated that the longitudinal expansion of leaves occurred allometrically against the latitudinal direction. For the same population, we found two different types of variation patterns with all 130 significant SNPs (Figures 4A,B); these showed that leaf shape differences were narrow vs. broad, and long-narrow vs. short-broad. For example, in comparison with *AA* and *AG*, the *GG* genotype at the SNP within the *PIN1* gene displayed the longest leaf axis and the narrowest leaf width (Figure 4A). The *TT*, *TG*, and *GG* genotypes with SNPs located at *CDK-like* genes only showed differences in width (Figure 4B). We classified these as “leaf length” and “leaf width” QTLs.

By dissecting shape pattern, along with the aforementioned “leaf length” QTL and “leaf width” QTL, two additional types of leaf shape divergence patterns were revealed in the low-altitude population (Figures 4C,D). Using the SNP at 7,547,290 bp of chromosome 1 as an example, with the segregating genotypes of *CC* and *CG*, both were found to have an identical shape feature at the blade base, and an equal length of leaf axis. Nonetheless, *CG* showed an enlarged margin edge at the middle-upper section of the leaf (Figure 4C); therefore, the expression of the two genotypes resulted in the “blade tip” QTL. Because genotypes with the “tip” QTL also showed an identical length axis on the leaves, segregating genotypes shared the position where the broadest blade region was located. Traditional methods cannot identify this type of leaf shape QTL. For the intercross QTL peak within chromosome 16, the closely linked SNP collection that included the positions of 1,282,251, 1,282,300, 1,282,031, and 1,285,142 bp showed a “blade base” QTL (Figure 4D). Although the “blade base” QTL belonged to one class of the “width” QTL, it was characterized by a perfectly consistent contour of the blade tip and the differentiated outline of the leaf base among genotypes. The new identification of “blade tip” and “blade base” QTLs of low-altitude provenance differentiated and enriched the pool of shape morphology variation.

### Altitudinal Expression of Shape QTL

**FIGURE 5:**
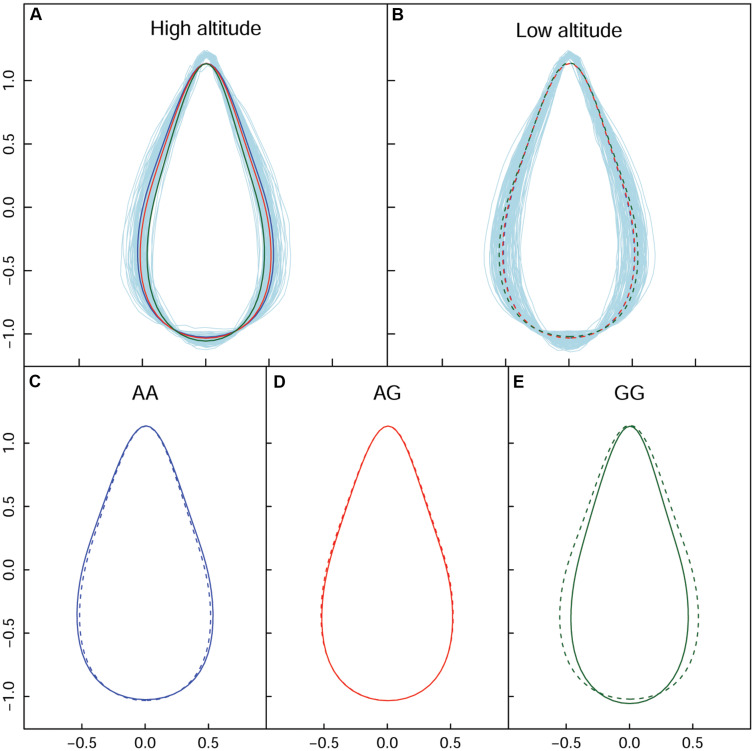
Impact of allelic expression of QTL on the *PIN1* gene. **(A,B)** Are the shape difference showed by the three genotypes respectively in high and low altitude. **(C–E)** Are the genotype-specific shape comparison for the two altitudes. Solid and broken lines indicate shape from high and low altitude.

In terms of a quantifiable leaf shape index, shape area and width, particular genotypes that incurred significant differences between the two populations are listed in Supplementary Table S3. From the union set of significant SNPs from the two altitude populations, 68 showed a change in leaf width of at least 10%, with maximum change of 24.52%. We used the SNP in the *PIN1* gene to explain the genetic effect of leaf shape pattern, because this SNP was significant at high altitude (Figure 5A), but was not significant at low altitude (Figure 5B). Both *AA* (Figure 5C) and *GA* (Figure 5D) tended to be stable and similar to each other, regardless of altitude; both dramatically differed from *GG* (Figure 5E). The reconstructed leaf shape that resulted from EF parameters enabled visualization of the components of leaf shape that differed among the three genotypes. Notably, comparison of leaf shape effects between high- and low-altitude populations revealed that *GG* considerably altered leaf shape; at low altitude, the width increase was 18.23% and the area increase was 16.40%. The expression difference with *GG* revealed that it caused 0.143% of heritability for controlling leaf shape at high altitude. Specifically, we identified reductions of leaf width and leaf area with the genes of *PIN1*, *CDK-like*, *IIIC63*, and *Protein kinase* at high altitude. For SNPs within other functional genes (*e.g., LRR kinase*, *NAP5*, *SCL1*, *BHLH76*), their genetic effects on shape are detailed in Table 2. Overall, there was a clear interaction between QTL and altitude.

**TABLE 2:** Genetic effects of SNPs within annotated genes.

Scaffold	Position	Gene	Full name	Gene ID	Allele	High-altitude population		Low-altitude population		Difference (%)	
						Area	Width	Area	Width	Area	Width
1	22082784	*CIPK3*	*CBL-INTERACTING PROTEIN KINASE 3*	POPTR_0019s08760	GG	1.677	1.107	1.598	1.054	4.96	5.05
1	28403445	*SAG101*	*SENESCENCE-ASSOCIATED CARBOXYLESTERASE 101*	POPTR_0001s29790	AG	1.760	1.169	1.644	1.085	7.04	7.72
1	43033561	*CRPK1*	*COLD-RESPONSIVE PROTEIN KINASE 1*	POPTR_0001s42950	AG	1.66	1.104	1.585	1.044	5.27	5.69
2	9660163	*AR781*	*AR781*	POPTR_0002s13000	TT	1.750	1.169	1.625	1.070	7.75	9.30
2	6092252	*DIS3L2*	*DIS3-like EXONUCLEASE 2*	POPTR_0002s08690	GG	1.823	1.212	1.598	1.051	14.08	15.33
4	22733081	*LRR kinase*	*LEUCINE RICH REPEAT kinase*	POPTR_0004s24030	TC	1.723	1.136	1.547	1.014	11.38	12.09
12	14701685	*LAP1*	*PROTEIN LAP1*	POPTR_0012s15030	CC	1.656	1.096	1.601	1.057	3.43	3.72
14	5416939	*COP11*	*COP9 signalosome complex subunit 1*	POPTR_0014s07160	AT	1.733	1.138	1.644	1.084	5.39	4.92
15	4747203	*PIN1*	*PIN-FORMED1*	POPTR_0015s04570	GG	1.468	0.964	1.610	1.075	9.64	11.46
15	8379891	*CDK-like*	*CYCLIN-DEPENDENT KINASE-LIKE*	POPTR_0015s07250	TT	1.475	0.970	1.530	1.007	3.75	3.90
18	9890878	*CAK1AT*	*CDK-ACTIVATING KINASE*	POPTR_0018s09000	TT	1.682	1.117	1.611	1.065	4.41	4.89
379	2531	*Stress-antifung*	*Salt stress response/antifungal*	POPTR_0379s00200	TC	1.701	1.129	1.615	1.069	5.33	5.64
1	36327299	*NAP5*	*NON-INTRINSIC ABC PROTEIN 5*	POPTR_0001s37340	TT	1.610	1.059	1.478	0.954	8.93	11.03
1	35770384	*JMT*	*JASMONIC ACID CARBOXYL METHYLTRANSFERASE*	POPTR_0001s36950	CC	1.619	1.063	1.444	0.942	12.05	12.90
2	21186580	*BHLH76*	*TRANSCRIPTION FACTOR BHLH76*	POPTR_0002s23650	TT	1.656	1.096	1.455	0.942	13.84	16.38
2	9487450	*Protein kinase*	*PROTEIN KINASE FAMILY PROTEIN*	POPTR_0002s12740	GG	1.656	1.096	1.455	0.942	13.84	16.38
3	18093953	*IIB*	*TRANSCRIPTION FACTOR IIB*	POPTR_0003s19330	GG	1.581	1.044	1.506	0.987	4.94	5.79
5	20022064	*SCL1*	*SCARECROW-LIKE1*	POPTR_0005s20890	CT	1.642	1.081	1.564	1.031	4.97	4.86
6	10067699	*EIN2*	*ETHYLENE-INSENSITIVE PROTEIN 2*	POPTR_0006s12900	AG	1.596	1.049	1.542	1.011	3.51	3.79
7	152674	*IIIC63*	*TRANSCRIPTION FACTOR IIIC63*	POPTR_0007s00430	TT	1.620	1.069	1.551	1.023	4.43	4.46
10	11244085	*Remorin*	*Remorin family protein*	POPTR_0010s10830	AA	1.609	1.058	1.402	0.902	14.74	17.28
11	17887288	*JMJ18*	*JUMOJI DOMAIN-CONTAINING PROTEIN 18*	POPTR_0011s15840	GG	1.633	1.080	1.523	0.999	7.22	8.03
16	13960977	*PIN-LIKES*	*PIN-FORMED LIKES*	POPTR_0016s14840	GG	1.642	1.086	1.517	1.001	8.23	8.48

## Discussion

The morphology of a leaf constitutes a significant component of ‘ideal plant type’ (Mock and Pearce, 1975). In accordance with Fick’s law, which specifies the substance diffusion rule, leaf shape affects substance exchange between the leaf and the outside world. Energy exchange on the blade surface can be equally affected by shape (Schuepp, 1993; Nicotra et al., 2008; Xu et al., 2009). This indicates that leaf shape plays an important role in maintaining plant function, as well as in the evolution of a plant’s environmental adaptability. Changes in photo-protective strategies, physiology, photosynthetic metabolism, and anatomical structures of leaves have been studied with respect to altitude gradients over long periods (Tranquillini, 1964; Zhang et al., 2017). Because of the potential evolutionary forces contributing to speciation, the mechanisms by which altitude adaptation is altered through the genetic architecture of organ shape have become central issues in morphological biology (Chitwood and Sinha, 2016).

Altitude is an exceedingly important environmental factor that influences evolutionary changes. In this study, through common garden test, we investigated genetic effects on leaf morphology using natural population comprising high- and low-altitude samples. *Q_ST_ – F_ST_* contrast showed three positive value with leaf length, leaf width and leaf area, suggesting the potential existence of local adaptation that *P*. *szechuanica* experienced, although *F*_ST_ revealed a very low degree of genetic divergence which can be ignored totally. Our results of shape dissection differed from studies of phenotypic plasticity, in which an organism alters its phenotype in response to environmental change (Schlichting, 1986). Given the two contrasting altitudes, the phenotypic variations we observed reflected the effects of the historical accumulation on leaf shape. It provides a new method to understand how altitude alters plant ecological adaptability to affect leaf shape through gene action and helps dissect the mechanisms of ecological adaptation in the context of ecological evolutionary developmental biology (Premoli et al., 2007).

Given on the accumulative heritability of shape area, width and length, the large difference between length and the other two traits (18.38% *versus* 6.916% and 0.263%) had suggested a stable expression of length on leaf axis, further indicating the easily changable trait of leaf width and leaf area relatively. Using the EF-based shape association model, our study successfully identified key genes for leaf shape in *P*. *szechuanica*. A total of 130 and 131 significant shape SNPs, respectively, for high- and low-altitude populations in the Qinghai-Tibetan plateau. Via annotation, *PIN1* was found as a known shape determination, its encoded auxin efflux carrier was involved in the maintenance of auxin gradients (Wenzel et al., 2010). *PIN-LIKES*, members within the same family, are similar to the *PIN* auxin transporter (Barbez et al., 2012). *NAP5*, the gene that encodes non-intrinsic ABC protein 5, is responsible for the transmembrane transport of auxin (Verrier et al., 2008). The exposures of these known shape genes and the class of auxin transporter gene contributed to the reliability of our leaf shape mapping results. Among them, *PPR2* and *Pkinase* were common regulatory genes, indicating that their shape regulation mechanism partially overlapped at both altitudes. Specific to low altitude, *EIN2* was found to negatively regulate miR164A, B, and C, thereby affecting leaf senescence in Kim et al. (2014) study. *JMJ18*, encoding a novel JmjC domain-containing histone H3K4 demethylase (a homologous member of *JMJ16* within the *JMJ* family), showed a leaf senescence repression role in *Arabidopsis* (Liu et al., 2019).

For the altitude-specific variation of leaf morphology, one possible explanation might involve the change of jasmonic acid (JA) regulated by senescence pathway. Since *JMJ18* and *JMT* were found in JA pathway to cause JA-mediated senescence, Wingler et al. (2015) reported a statistically significant negative correlation between higher JA and lower chlorophyll contents, but only when both temperature treatments were combined. Therefore, the connection between altitude and shape variation might be established through *JMJ* and *JMT* gene, because temperature is one of the main factor comprised in altitude. The significance of these leaf-senescence genes indicated that senescence-related physiology may contribute to the regulation of leaf shape at low altitude.

Interestingly, through the annotation of *Stress-antifung*, *CIPK3* and *CRPK1*, their importance with Stress-related functionality and expression in response to stress stimulation in high-altitude, *e.g.*, the role of the stress-antifung gene in mediating defense response, as well as the role of *LAP1*, which functions to alleviate stress-induced damage (Scranton et al., 2012). The role of *CIPK3* in response to abscisic acid, cold, drought, high salt and wounding condition might reflect its significance for plant growth in high-altitude, where these condition might co-existed (Sanyal et al., 2017). Similarly, the significant result with cold-stress responsive role of *CRPK1* might also represent one special defense to cause shape variation (Liu et al., 2017). *CAK1AT* was detected in the high-altitude population; this gene is annotated as a regulator of cell differentiation through control of CDK activity (Takatsuka et al., 2015). Similar to a *CDK-like* gene, *CAK1AT* was also included in the list of significant genes. Our identification of other cell-differentiation-related genes, such as *SCL1*, in the low-altitude population implied a potential interplay between two modules, *i.e*., the organ-wide growth-reflecting differentiation and the local cell division involving leaf edge (Kierzkowski et al., 2019). Balance among these may generate the morphological diversity of leaves in high-altitude populations.

Through classification of “leaf length,” “leaf width,” “blade tip” and “blade base” genes, we discovered the exclusive expression of “blade tip” and “blade base” genes in the low-altitude population, which suggested that low altitude can assist a more tolerant environment for developing diversified leaf shapes. It also indicated the potential for greater plasticity among trees that grew in the low-altitude environment. Environmental factors in Qinghai-Tibetan plateau combines to form a unique case to study adaptive evolution (Li and Fang, 1999). By inspecting the impact of altitude on the allelic expression of leaf shape genes, the study revealed the complexity of the gene-by-altitude interaction, which denotes one of the G × E phenomena in a biological system (El-Soda et al., 2014). A comparison of the genotypic effect on leaf shape between distinct altitudes had minuted what shape aspects of QTLs that would be evolutionarily different. For instance, *GG* within the SNP of *PIN1* produce a longer leaf axis and narrower lamina for high-altitude populations, which was consistent with the need for narrower shapes in high-altitude populations to prevent water loss and to reduce allocation of leaf mass caused by high light intensity (Gregory-Wodzicki, 2000; Royer et al., 2009; Peppe et al., 2011). The combination of harsh environmental stresses in Qinghai-Tibetan plateau potentially subjects individuals to a series of adaptive changes. Thus, in the context of adaptation, the study provided the opportunity for in-depth exploration of the relationships between leaf shape diversity and biological adaptation; these aspects are linked through particular genes, metabolic clues, and biochemical pathways that underlie these fundamental issues of adaptation.

## Data Availability Statement

SNP typing data was uploaded to European Variation Archive (EVA) database, with a project ID of PRJEB36028. The web link is https://www.ebi.ac.uk/ena/data/view/PRJEB36028.

## Author Contributions

MY performed the data analyses and wrote the manuscript. LJ derived the model. PG and XZ participated in the field management of experimental materials and led the phenotype investigation and data collection. RW conceived of the idea of the overall investigation.

## Conflict of Interest

The authors declare that the research was conducted in the absence of any commercial or financial relationships that could be construed as a potential conflict of interest.

## Footnotes

1 https://ftp.ncbi.nlm.nih.gov/genomes/refseq/plant/Populus_trichocarpa/latest_assembly_versions/GCF_000002775.4_Pop_tri_v3/GCF_000002775.4_Pop_tri_v3_genomic.fna.gz

2 http://bio-bwa.sourceforge.net/bwa.shtml

3 http://www.htslib.org

4 https://www.ebi.ac.uk/eva

## Supplementary Material

FIGURE S1 Sampling sites, showing distributions of high- and low-altitude populations in Sejila mountain in the Qinghai-Tibetan plateau.

FIGURE S2 Population structures for high- and low-altitude populations. **(A,B)** Represent the likelihood change over *K*s and population structures at high and low altitudes, respectively. Different colors denote distinct sub-populations; bar heights with different colors represent membership probabilities of individuals.

FIGURE S3 Fitting of leaf shape using different harmonic orders of Elliptic Fourier (EF) parameters. Gray color denotes the true average leaf, while reconstructed shapes using EF parameters are shown with thick green lines.

TABLE S1 Detailed annotations with identification of SNPs significant in the regulation of shape variation in high-altitude populations.

TABLE S2 Detailed annotations with identification of SNPs significant in the regulation of shape variation in low-altitude populations.

TABLE S3 Comparison of QTL expressions between high and low altitudes.

FILE S1 The R script for GWAS of leaf shape.
