# Supplementary figures and images for: Identification of Quantitative Trait Loci for Altitude Adaptation of Tree Leaf Shape With Populus szechuanica in the Qinghai-Tibetan Plateau
Source: Front Plant Sci. 2020 May 27;11:632. doi: 10.3389/fpls.2020.00632 (PMC7267013; doi:10.3389/fpls.2020.00632)

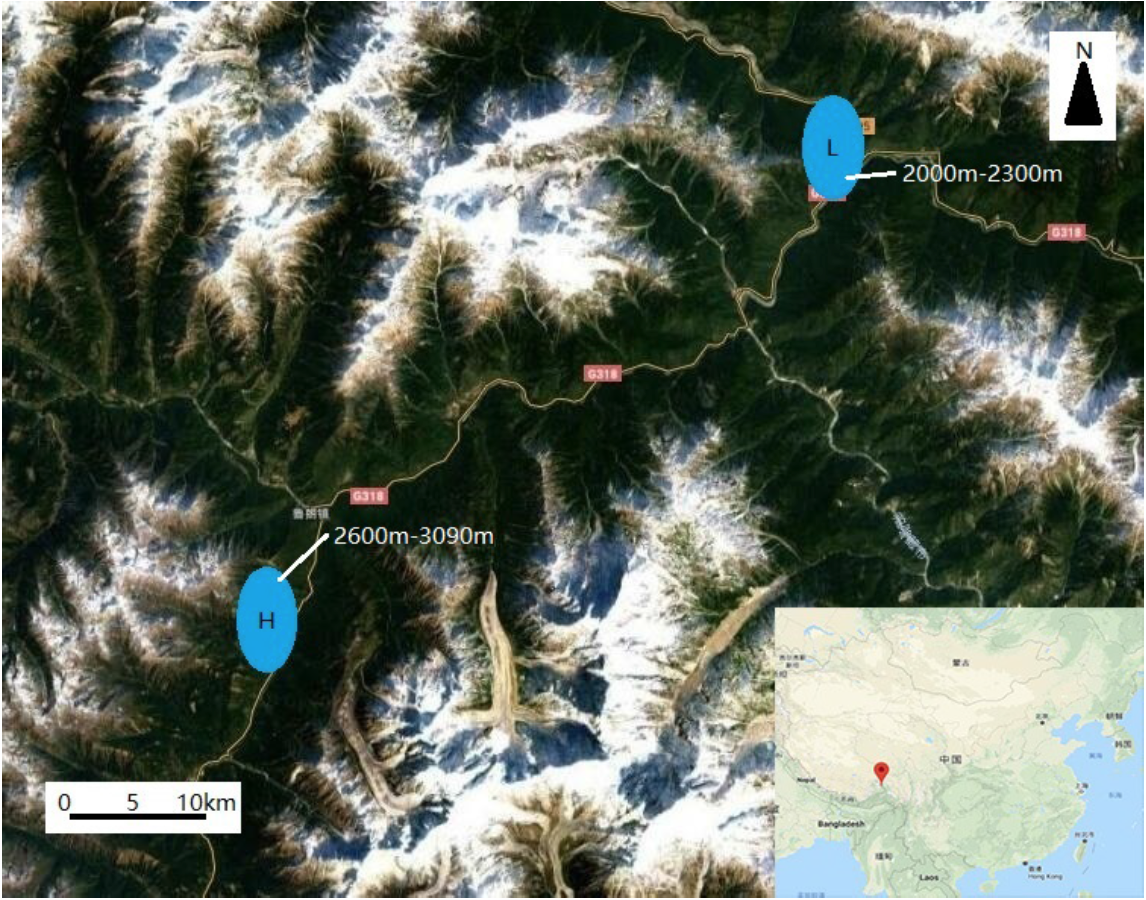

Supplement: FIGURE S1 — Sampling sites, showing distributions of high- and low-altitude populations in Sejila mountain in the Qinghai-Tibetan plateau. [file Image_1.TIF]

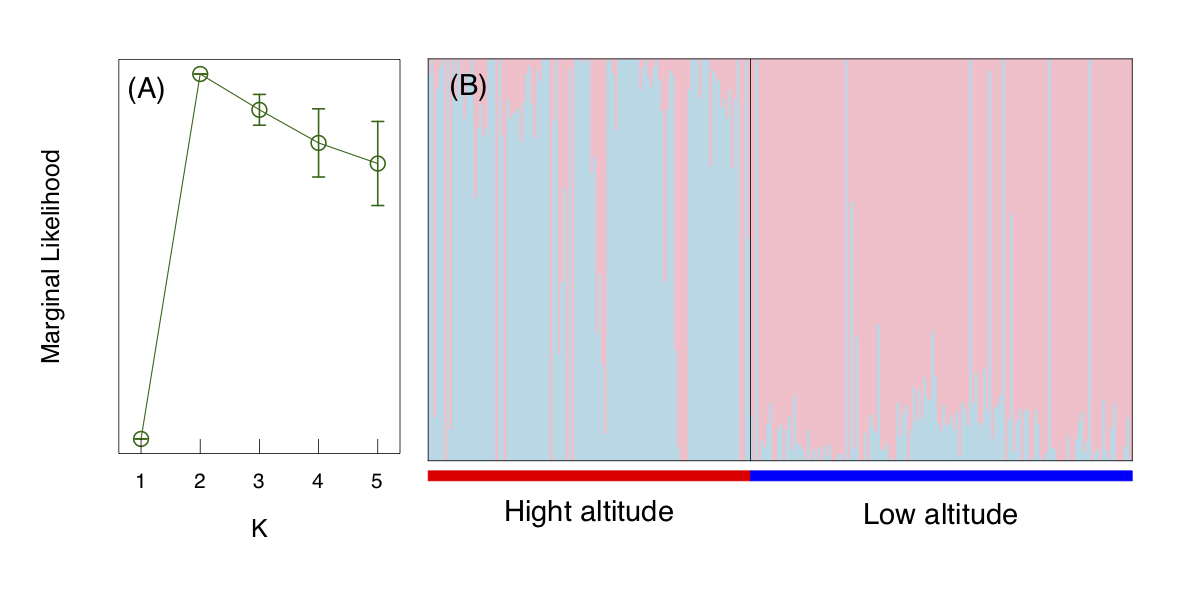

Supplement: FIGURE S2 — Population structures for high- and low-altitude populations. (A,B) Represent the likelihood change over Ks and population structures at high and low altitudes, respectively. Different colors denote distinct sub-populations; bar heights with different colors represent membership probabilities of individuals. [file Image_2.tiff]

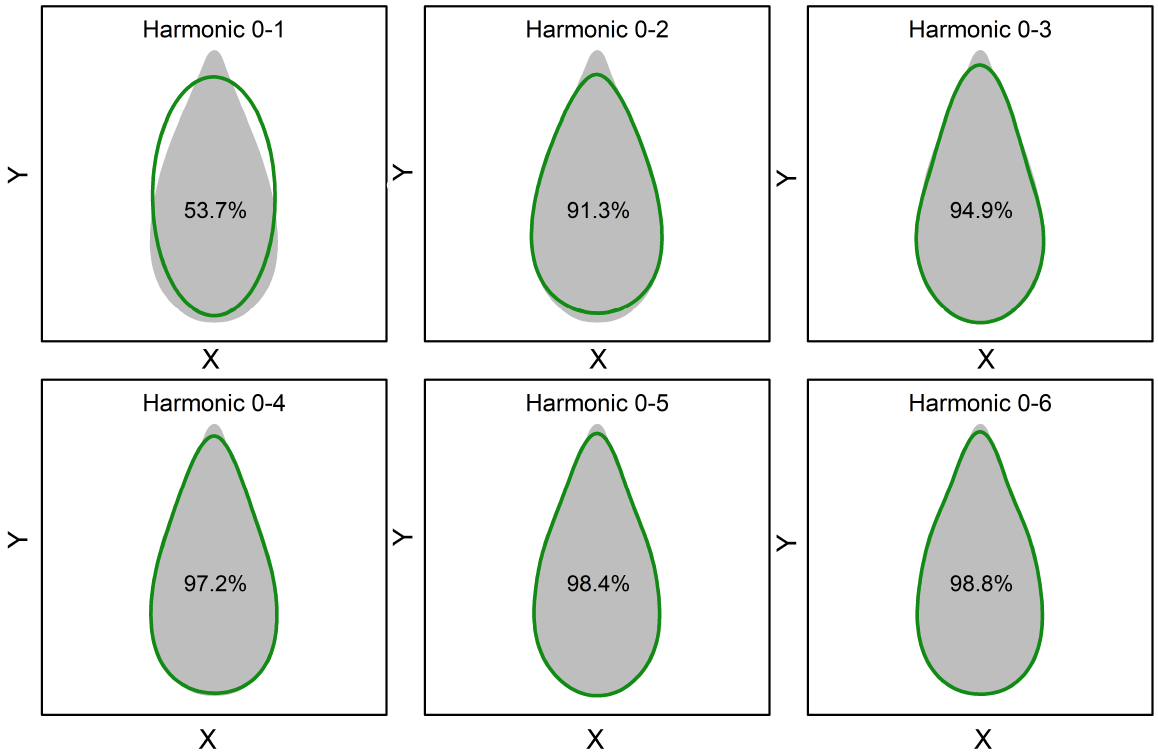

Supplement: FIGURE S3 — Fitting of leaf shape using different harmonic orders of Elliptic Fourier (EF) parameters. Gray color denotes the true average leaf, while reconstructed shapes using EF parameters are shown with thick green lines. [file Image_3.tif]
